# Supplementary material for: A Critical View of the Application of the APEX Software (Aqueous Photochemistry of Environmentally-Occurring Xenobiotics) to Predict Photoreaction Kinetics in Surface Freshwaters
Source: Molecules. 2019 Dec 18;25(1):9. doi: 10.3390/molecules25010009 (PMC7017383; doi:10.3390/molecules25010009)
Supplement: Supplementary file 1 [file molecules-25-00009-s001.zip › 3.2.4_gcc-4.4.0.zip!/3.2.4_gcc-4.4.0/3.2.4_gcc-4.4.0/doc/octave/html/interpreter/Creating-Sparse-Matrices.html]

Creating Sparse Matrices - Untitled


Next: Information,
Previous: Storage of Sparse Matrices,
Up: Basics


---

#### 21.1.2 Creating Sparse Matrices

There are several means to create sparse matrix.

Returned from a function: There are many functions that directly return sparse matrices. These include speye, sprand, diag, etc. Constructed from matrices or vectors: The function sparse allows a sparse matrix to be constructed from three vectors representing the row, column and data. Alternatively, the function spconvert uses a three column matrix format to allow easy importation of data from elsewhere. Created and then filled: The function sparse or spalloc can be used to create an empty matrix that is then filled by the user From a user binary program: The user can directly create the sparse matrix within an oct-file.

There are several basic functions to return specific sparse
matrices. For example the sparse identity matrix, is a matrix that is
often needed. It therefore has its own function to create it as
`speye (`n`)` or `speye (`r`,` c`)`, which
creates an n-by-n or r-by-c sparse identity
matrix.

Another typical sparse matrix that is often needed is a random distribution
of random elements. The functions sprand and sprandn perform
this for uniform and normal random distributions of elements. They have exactly
the same calling convention, where `sprand (`r`,` c`,` d`)`,
creates an r-by-c sparse matrix with a density of filled
elements of d.

Other functions of interest that directly create sparse matrices, are
diag or its generalization spdiags, that can take the
definition of the diagonals of the matrix and create the sparse matrix
that corresponds to this. For example

```
     s = diag (sparse(randn(1,n)), -1);
```

creates a sparse (n+1)-by-(n+1) sparse matrix with a single
diagonal defined.

— Function File: [b, c] = **spdiags** (a)  
— Function File: b = **spdiags** (a, c)  
— Function File: b = **spdiags** (v, c, a)  
— Function File: b = **spdiags** (v, c, m, n)  
> A generalization of the function `diag`. Called with a single
> input argument, the non-zero diagonals c of A are extracted.
> With two arguments the diagonals to extract are given by the vector
> c.
>
> The other two forms of `spdiags` modify the input matrix by
> replacing the diagonals. They use the columns of v to replace
> the columns represented by the vector c. If the sparse matrix
> a is defined then the diagonals of this matrix are replaced.
> Otherwise a matrix of m by n is created with the
> diagonals given by v.
>
> Negative values of c represent diagonals below the main
> diagonal, and positive values of c diagonals above the main
> diagonal.
>
> For example
>
> ```
>           spdiags (reshape (1:12, 4, 3), [-1 0 1], 5, 4)
>           =>    5 10  0  0
>                 1  6 11  0
>                 0  2  7 12
>                 0  0  3  8
>                 0  0  0  4
> ```

— Function File: y = **speye** (m)  
— Function File: y = **speye** (m, n)  
— Function File: y = **speye** (sz)  
> Returns a sparse identity matrix. This is significantly more
> efficient than `sparse (eye (`m`))` as the full matrix
> is not constructed.
>
> Called with a single argument a square matrix of size m by
> m is created. Otherwise a matrix of m by n is
> created. If called with a single vector argument, this argument
> is taken to be the size of the matrix to create.

— Function File: y = **spfun** (f,x)  
> Compute `f(`x`)` for the non-zero values of x.
> This results in a sparse matrix with the same structure as
> x. The function f can be passed as a string, a
> function handle or an inline function.

— Mapping Function: **spmax** (x, y, dim)  
— Mapping Function: [w, iw] = **spmax** (x)  
> This function has been deprecated. Use `max` instead.

— Mapping Function: **spmin** (x, y, dim)  
— Mapping Function: [w, iw] = **spmin** (x)  
> This function has been deprecated. Use `min` instead.

— Function File: y = **spones** (x)  
> Replace the non-zero entries of x with ones. This creates a
> sparse matrix with the same structure as x.

— Function File: **sprand** (m, n, d)  
— Function File: **sprand** (s)  
> Generate a random sparse matrix. The size of the matrix will be
> m by n, with a density of values given by d.
> d should be between 0 and 1. Values will be uniformly
> distributed between 0 and 1.
>
> Note: sometimes the actual density may be a bit smaller than d.
> This is unlikely to happen for large really sparse matrices.
>
> If called with a single matrix argument, a random sparse matrix is
> generated wherever the matrix S is non-zero.
>
>
>
> **See also:** sprandn.

— Function File: **sprandn** (m, n, d)  
— Function File: **sprandn** (s)  
> Generate a random sparse matrix. The size of the matrix will be
> m by n, with a density of values given by d.
> d should be between 0 and 1. Values will be normally
> distributed with mean of zero and variance 1.
>
> Note: sometimes the actual density may be a bit smaller than d.
> This is unlikely to happen for large really sparse matrices.
>
> If called with a single matrix argument, a random sparse matrix is
> generated wherever the matrix S is non-zero.
>
>
>
> **See also:** sprand.

— Function File: **sprandsym** (n, d)  
— Function File: **sprandsym** (s)  
> Generate a symmetric random sparse matrix. The size of the matrix will be
> n by n, with a density of values given by d.
> d should be between 0 and 1. Values will be normally
> distributed with mean of zero and variance 1.
>
> Note: sometimes the actual density may be a bit smaller than d.
> This is unlikely to happen for large really sparse matrices.
>
> If called with a single matrix argument, a random sparse matrix is
> generated wherever the matrix S is non-zero in its lower
> triangular part.
>
>
>
> **See also:** sprand, sprandn.

The recommended way for the user to create a sparse matrix, is to create
two vectors containing the row and column index of the data and a third
vector of the same size containing the data to be stored. For example

```
       ri = ci = d = [];
       for j = 1:c
         ri = [ri; randperm(r)(1:n)'];
         ci = [ci; j*ones(n,1)];
         d = [d; rand(n,1)];
       endfor
       s = sparse (ri, ci, d, r, c);
```

creates an r-by-c sparse matrix with a random distribution
of n (<r) elements per column. The elements of the vectors
do not need to be sorted in any particular order as Octave will sort
them prior to storing the data. However, pre-sorting the data will
make the creation of the sparse matrix faster.

The function spconvert takes a three or four column real matrix.
The first two columns represent the row and column index respectively and
the third and four columns, the real and imaginary parts of the sparse
matrix. The matrix can contain zero elements and the elements can be
sorted in any order. Adding zero elements is a convenient way to define
the size of the sparse matrix. For example

```
     s = spconvert ([1 2 3 4; 1 3 4 4; 1 2 3 0]')
     => Compressed Column Sparse (rows=4, cols=4, nnz=3)
           (1 , 1) -> 1
           (2 , 3) -> 2
           (3 , 4) -> 3
```

An example of creating and filling a matrix might be

```
     k = 5;
     nz = r * k;
     s = spalloc (r, c, nz)
     for j = 1:c
       idx = randperm (r);
       s (:, j) = [zeros(r - k, 1); ...
             rand(k, 1)] (idx);
     endfor
```

It should be noted, that due to the way that the Octave
assignment functions are written that the assignment will reallocate
the memory used by the sparse matrix at each iteration of the above loop.
Therefore the spalloc function ignores the nz argument and
does not preassign the memory for the matrix. Therefore, it is vitally
important that code using to above structure should be vectorized
as much as possible to minimize the number of assignments and reduce the
number of memory allocations.

— Loadable Function: FM = **full** (SM)  
> returns a full storage matrix from a sparse, diagonal, permutation matrix or a range.
>
>
>
> **See also:** sparse.

— Function File: s = **spalloc** (r, c, nz)  
> Returns an empty sparse matrix of size r-by-c. As Octave
> resizes sparse matrices at the first opportunity, so that no additional
> space is needed, the argument nz is ignored. This function is
> provided only for compatibility reasons.
>
> It should be noted that this means that code like
>
> ```
>           k = 5;
>           nz = r * k;
>           s = spalloc (r, c, nz)
>           for j = 1:c
>             idx = randperm (r);
>             s (:, j) = [zeros(r - k, 1); rand(k, 1)] (idx);
>           endfor
> ```
>
> will reallocate memory at each step. It is therefore vitally important
> that code like this is vectorized as much as possible.
>
>
>
> **See also:** sparse, nzmax.

— Loadable Function: s = **sparse** (a)  
— Loadable Function: s = **sparse** (i, j, sv, m, n, nzmax)  
— Loadable Function: s = **sparse** (i, j, sv)  
— Loadable Function: s = **sparse** (i, j, s, m, n, "unique")  
— Loadable Function: s = **sparse** (m, n)  
> Create a sparse matrix from the full matrix or row, column, value triplets.
> If a is a full matrix, convert it to a sparse matrix representation,
> removing all zero values in the process.
>
> Given the integer index vectors i and j, a 1-by-`nnz` vector
> of real of complex values sv, overall dimensions m and n
> of the sparse matrix. The argument `nzmax` is ignored but accepted for
> compatibility with matlab. If m or n are not specified their
> values are derived from the maximum index in the vectors i and j
> as given by m `= max (`i`)`, n `= max (`j`)`.
>
> **Note**: if multiple values are specified with the same
> i, j indices, the corresponding values in s will
> be added.
>
> The following are all equivalent:
>
> ```
>           s = sparse (i, j, s, m, n)
>           s = sparse (i, j, s, m, n, "summation")
>           s = sparse (i, j, s, m, n, "sum")
> ```
>
> Given the option "unique". if more than two values are specified for the
> same i, j indices, the last specified value will be used.
>
> `sparse(`m`,` n`)` is equivalent to
> `sparse ([], [], [],` m`,` n`, 0)`
>
> If any of sv, i or j are scalars, they are expanded
> to have a common size.
>
>
>
> **See also:** full.

— Function File: x = **spconvert** (m)  
> This function converts for a simple sparse matrix format easily
> produced by other programs into Octave's internal sparse format. The
> input x is either a 3 or 4 column real matrix, containing
> the row, column, real and imaginary parts of the elements of the
> sparse matrix. An element with a zero real and imaginary part can
> be used to force a particular matrix size.

The above problem of memory reallocation can be avoided in
oct-files. However, the construction of a sparse matrix from an oct-file
is more complex than can be discussed here, and
you are referred to chapter Dynamically Linked Functions, to have
a full description of the techniques involved.
